# TNF-α Levels Are Increased in Patients with Subjective Cognitive Impairment and Are Negatively Correlated with β Amyloid-42

**DOI:** 10.3390/antiox13020216

**Published:** 2024-02-08

**Authors:** Sara Serafini, Gabriella Ferretti, Paola Monterosso, Antonella Angiolillo, Alfonso Di Costanzo, Carmela Matrone

**Affiliations:** 1 Unit of Pharmacology, Department of Neuroscience, Faculty of Medicine, University of Naples Federico II, Via Pansini 5, 80131 Naples, Italy; sara.serafini@unina.it (S.S.); gabriella.ferretti@unina.it (G.F.); paola.monterosso@studenti.unina.it (P.M.); 2 Department of Medicine and Health Sciences, Center for Research and Training in Aging Medicine, University of Molise, 86100 Campobasso, Italy; antonella.angiolillo@unimol.it

**Keywords:** Alzheimer’s disease, TNF-α, neuroinflammation, biomarkers, subjective cognitive impairment, mild cognitive impairment, amyloid beta, neurodegeneration, Fyn, APP Tyr682 phosphorylation

## Abstract

The role of tumor necrosis factor-α (TNF-α) in Alzheimer’s disease (AD) has recently become a topic of debate. TNF-α levels increase in the blood of patients with AD, and amyloid beta (Aβ) plaques contain TNF-α deposits. The therapeutic efficacy of blocking TNF-α in patients with AD remains controversial as it is mostly based on preclinical studies. Thus, whether and how TNF-α contributes to amyloidogenic processes in AD is still an open question to be addressed. We analyzed plasma TNF-α and Aβ42 levels in patients with subjective cognitive impairment (SCI), mild cognitive impairment (MCI), and AD, and in healthy volunteers (HLT). In addition, we performed correlation analysis to evaluate whether changes in plasma TNF-α levels correlate with cognitive decline, Aβ42 levels, age, and BMI, which are all factors considered to contribute to or predispose individuals to AD. We found that TNF-α and Aβ42 plasma levels were higher in patients with AD than in HLT individuals. High TNF-α levels were also observed in patients with SCI, in whom TNF-α and Aβ42 levels were negatively correlated. Notably, TNF-α did not affect the amyloidogenic pathway in human microglial cultures exposed to 48 h of incubation, although it did trigger neuroinflammatory processes. These results imply that high TNF-α levels are more likely to be a clinical condition linked to AD than are direct contributors. Nonetheless, elevated levels of TNF-α in early-stage patients, like those with SCI and MCI, may provide a distinguishing feature for identifying clinical profiles that are at risk of having a poorer outcome in AD and could benefit from tailored therapies.

## 1. Introduction

Alzheimer’s disease (AD) is a degenerative brain disorder characterized by cognitive decline and two primary abnormalities: the accumulation of extracellular amyloid beta (Aβ) plaques and intracellular neurofibrillary tangles [1]. In recent years, many studies have highlighted the importance of the immune response in the brain and proposed neuroinflammation as the third primary feature of AD [2,3]. Accordingly, Aβ deposits in the brain can lead to the activation of the immune response and recruitment of brain-resident macrophages and microglia, which worsens both Aβ and tau pathologies and promotes disease progression [4].

Tumor necrosis factor alpha (TNF-α) is a pro-inflammatory cytokine expressed by microglia, astrocytes, and neurons in the brain and mononuclear cells in peripheral blood circulation. Its production increases under several pathological conditions, including AD [5,6]. 

TNF-α has been found to accumulate within Aβ plaques in the postmortem brains of patients with AD and in the plasma and cerebrospinal fluid (CSF) [7,8], thus triggering disease progression and accelerating cognitive decline [9]. In addition, TNF-α polymorphisms linked to increased TNF-α levels have been detected in patients with late-onset AD [10,11,12]. Of note, patients with rheumatoid arthritis, psoriasis, and lupus treated with anti-inflammatory medications targeting TNF-α show a lower risk of developing AD [7,8,9], and the TNF-α inhibitors infliximab and etanercept significantly reduce cognitive deficits in patients with AD upon intrathecal and perispinal administration, respectively [7,9,13]. 

Studies in mice modeling AD indicate that the removal of TNF receptor 1 (TNFR1) or the use of TNF-α inhibitors reduces the presence of Aβ [5]. In addition, peripheral TNF-α modulates amyloid pathology by regulating blood-derived immune cell trafficking into the brain in double-transgenic mice with combined amyloid pathology and human TNF-α overexpression [14]. Interestingly, transient expression of TNF-α in the hippocampus decreases amyloid deposition [15], while chronic neuronal expression of TNF-α has been shown to worsen Aβ pathology [16], suggesting that TNF-α may have both beneficial and detrimental effects on AD depending on the timing and location of its expression. 

Additionally, TNF-α stimulates the excessive release of harmful reactive oxygen species (ROS) and nitrogen species (RNS) through the activation of nuclear factor kappa B (NF-κB) and the increase in inducible nitric oxide synthase (iNOS) expression in cellular and animal models of AD or dementia [17]. This is likely to be initiated in astrocytes and microglia but quickly propagates to neurons, where it activates a neuroinflammatory loop that results in neuronal toxicity and death [17,18,19].

Indeed, this dual role exerted by TNF-α, either in promoting oxidative stress or triggering peripheral as well as brain inflammation, makes it a noteworthy biomarker for AD. Notably, TNF-α-related inflammatory processes and oxidative stress significantly contribute to the progression of AD [19,20].

Despite an increasing number of preclinical studies and clinical trials pointing to TNF-α as a druggable target in AD, the direct causal relationship between the two remains to be confirmed. Recently, we found that TNF-α and Aβ42 levels were higher in patients with AD than in healthy controls (HLT). However, TNF-α increase was not associated with changes in Aβ42 levels or a decrease in the Mini Mental State Examination (MMSE) score [21]. In addition, metabolomic analysis of plasma from HLT participants or patients with AD suggested that the lipidomic profile of individuals with higher TNF-α levels was mostly associated with alterations in the triglyceride profile, whereas the increase in Aβ42 and decrease in MMSE scores correlated better with alterations in phosphatidylcholine and lysophosphatylcholine patterns [21].

In this study, we examined TNF-α levels in 34 healthy volunteers (HLT) and 99 patients, including 30 with subjective cognitive impairment (SCI) and 30 with mild cognitive impairment (MCI), which are considered preclinical stages that can evolve into AD [22], and 39 with AD (AD). Changes in TNF-α levels were examined in relation to cognitive decline, plasma Aβ42 levels, age, and BMI, which have previously been demonstrated to contribute to AD [23,24].

Our findings indicated that TNF-α levels were higher in patients with SCI and AD than in HLT controls. Patients with AD also showed higher TNF-α levels than those with MCI did. Similarly, Aβ42 plasma levels were higher in the AD group than those in the MCI and HLT groups. Notably, TNF-α levels correlated negatively with Aβ42 levels in patients with SCI and MCI but not in AD and HLT subjects. Interestingly, TNF-α exposure activates the inflammatory pathway in human healthy microglial cell cultures but does not affect the amyloidogenic pathway, by promoting amyloid precursor protein (APP) phosphorylation on the tyrosine (Tyr) 682 residue (APPpTyr682), which promotes Aβ42 production in neurons, fibroblasts, and blood mononuclear cells [21,25,26,27], and APP intracellular processing. In addition, we did not detect changes in Fyn tyrosine kinase phosphorylation, which is considered a key player in AD, triggering either APPpTyr682 [27] or tau protein phosphorylation on the Tyr residue [28]. This study underscores the significance of focusing on TNF-α-related pathways in patients in the initial stages of AD, with the aim of developing pharmacological interventions that tailor their individual clinical presentation.

## 2. Materials and Methods

### 2.1. Participants

Thirty-four cognitively healthy subjects (HLT), thirty patients with subjective cognitive impairment (SCI), thirty patients with mild cognitive impairment (MCI), and thirty-nine patients with AD (AD) were enrolled from the Center for Research and Training in Medicine for Aging at the University of Molise, Campobasso (Italy). Patients with AD were diagnosed according to the National Institute on Aging/Alzheimer’s Association (NIA-AA) criteria [18], fulfilled the criteria for “probable AD with documented decline”, and showed a Mini Mental State Examination (MMSE) score < 24 and Clinical Dementia Rating (CDR) score > 0.5. Patients with amnestic MCI who met the NIA-AA diagnostic criteria for MCI due to AD [29] had an MMSE score > 24 and a CDR of 0.5. Patients with SCI complained of a deterioration in their memory compared to earlier stages in life, had a score of 25 or more on the Memory Complaint Questionnaire (MAC-Q), and showed normal performance on memory tests. To summarize, MCI patients showed both subjective and objective memory impairment, SCI patients presented only memory complaints with a normal score on neuropsychological tests, and HLT patients showed neither subjective nor objective memory impairment. To rule out other potential causes of cognitive impairment, all participants underwent blood tests (including full blood count, erythrocyte sedimentation rate, urea and electrolytes, thyroid function, vitamin B12, and folate levels), and all patients with AD and MCI and 20 out of 30 subjects with SCI underwent brain imaging. Depression was also ruled out using the Geriatric Depression Scale—Short Form (GDS-SF) and participants with a GDS-SF score of 6 or more were excluded from the study. Patients receiving treatment with cerebro-active drugs underwent a washout period of at least 14 days before the assessment. Participants with a GDS-SF score ≥ 6 and individuals who consumed more than two standard drinks per day for females or three standard drinks per day for males, or were taking medication that could impact the central nervous system, were excluded from the study. The clinical and demographic characteristics of the four groups of participants (SCI, MCI, AD, and HLT) are shown in Table 1.

This study adhered to the ethical principles of the Declaration of Helsinki and the National and International Guidelines for Human Research. The Institutional Review Board (IRB) of the University of Molise approved this study (IRB Prot. N. 16/2020). The participants or caregivers provided written informed consent before enrolment in the study.

### 2.2. Blood Sampling

Approximately 6 mL blood was collected from each patient. Plasma was separated from peripheral blood mononuclear cells (PBMCs) immediately after collecting the blood samples and analyzed by ELISA. PBMCs were frozen and stored. Only the plasma samples were available for this study.

### 2.3. Enzyme-Linked Immunosorbent Assay (ELISA)

ELISA kits #CSB-E10684h and #CSB-E04740h (Cusabio, Houston, TX, USA) were used to assess Aβ42 and TNF-α plasma levels following the manufacturer’s instructions. Datasheets reported kit sensitivity of 0.078 ng/mL and 1.95 pg/mL for Aβ42 and TNFα, respectively. Values below the sensitivity of the kit were excluded from analysis.

### 2.4. Microglia Cell Culture

Human primary cultures of microglia (HMC3, 37,089–01) were purchased from Celprogen, Inc. (Benelux, NL, USA) and cultured in Dulbecco’s Modified Eagle’s medium (Sigma-Aldrich, Milan, Italy) following a previously described procedure [30,31].

Two inhibitors, etanercept (European Pharmacopeia Reference Standard, #Y0001969, CAS Number 185243-69-0) and infliximab (European Pharmacopeia Reference Standard, #Y0002047, CAS Number 185243-69-0), were used to inhibit the effects of TNF-α (Sigma-Aldrich, Milan, Italy, SRP3177) in microglial cell culture. The lowest concentrations of TNF-α, etanercept, and infliximab at which the highest pharmacological effect was achieved without causing cell death were evaluated (Figure S1A,D), following the indications provided by the supplier in datasheets.

In experiments reported in Figure S1A, microglial cells were incubated with TNF-α at concentrations ranging from 80 ng/mL to 240 ng/mL. The concentration of 120 ng/mL, which was the lowest concentration at which we assessed its effect on cell proliferation, was selected for all the experiments (Figure S1A). Figure S2B shows the number of DAPI-positive nuclei 24 h and 48 h after TNF-α exposure. Values were compared to those from control conditions (Figure S2A,D). Because significant differences in the number of microglial cells were detectable only after 48 h of incubation (Figure S2D), all experiments were performed at this time point. Additionally, the effect of repeated exposure to TNF-α was evaluated by exposing cells to TNF-α twice at t0 (24 h after plating) and t24 (after 24 h of incubation) after plating.

Etanercept (Eta, 1 μg/mL) and infliximab (Infl, 50 μg/mL) were added 24 h after TNF-α incubation and left in culture for an additional 24 h. The optimal concentration of inhibitors was assessed after a dose–response curve, as well as following the supplier indication (Figure S1C,D). Note that TNF-α and inhibitor solubilization and dilution were performed using the same growth media of microglial cells.

After 48 h of incubation with or without the inhibitors, microglial cells were processed for immunofluorescence (IF), immunoprecipitation (IP), and Western blot (WB) analyses.

### 2.5. Western Blot and Immunoprecipitation (IP)

Protein lysates were prepared by homogenization in RIPA buffer (Sigma-Aldrich, Milan, Italy) supplemented with protease inhibitors (Thermo Fisher Scientific, Milan, Italy). The protein concentration was determined using the Bradford assay. For each experimental condition, 30 μg of protein was loaded onto a 4–15% precast polyacrylamide gel (Bio-Rad Laboratories, Milan, Italy) under reducing conditions and transferred to PVDF membranes (Abcam, Cambridge, UK). Detection of the protein of interest was accomplished using a chemiluminescence method with Clarity Western ECL Substrate (Bio-Rad Laboratories, Milan, Italy). Digital quantification was performed through densitometric analysis of the immunoreactive bands using ImageLab 6.1.0 software (2020, Bio-Rad Laboratories, Milan, IT, USA).

For IP analysis, 100 μg of total protein was incubated with mouse anti-pTyr antibody (clone pTyr-100) magnetic bead conjugate (30 µg/100 µL, #8095, Cell Signalling Technology, DBA, Milan, Italy) following a previously described procedure [26,32]. After 48 h of incubation, the IP samples were washed in PBS and processed by WB using mouse anti-APP antibody, clone Y188 (1:1000, #ab32136, Abcam, Cambridge, UK). The following antibodies were used for WB analysis: rabbit anti-pan Fyn (1:1000, #4023 Cell Signaling Technology, DBA, Milan, Italy), rabbit anti-Src pTyr416 (1:1000, #2101, Cell Signaling Technology, DBA, Milan, Italy), mouse anti-APP, clone 22C11 (1:500, #MAB348, Merck/Chemicon, Milan, Italy), and mouse anti-β actin (1:20000, #A3854, Merck/Sigma-Aldrich, Milan, Italy).

### 2.6. Immunofluorescence

Microglial cells after 48 h exposure to TNF-α with or without inhibitors were fixed in 4% PFA-methanol free solution (ThermoFisher, Milan, Italy), and permeabilized with 0.05% Triton X-100 (Bio-Rad Laboratories, Milan, Italy) for 3–5 min at room temperature. The cells were then rinsed with DPBS, and incubated with the primary antibodies overnight at 4 °C. The primary antibodies used were mouse ant-Iba1 (1:1000, #66,827–1-Ig, Proteintech, DBA, Milan, Italy), rabbit anti-TMEM119 (1:1000, #66,948–1-Ig Proteintech, DBA, Milan, Italy), mouse anti-iNOS (1:1000, #18,985–1-AP Proteintech, DBA, Milan, Italy), and rabbit anti-TNF-α (1:1000, #3707; Cell Signaling Technology, DBA, Milan, Italy). Secondary antibodies conjugated to Alexa 488 (1:250, #A-11029, Invitrogen, Milan, Italy) or 594 (1:250, #R37117, Invitrogen, Milan, Italy) fluorochromes were used to detect the primary antibodies. DAPI (Fluoroshield Mounting Medium with DAPI, #Ab104139 Abcam, Cambridge, UK) was used to visualize the nuclei and count cells.

### 2.7. Image Acquisition and Processing

All images were acquired with an LSM700 AxioObserver laser scanning confocal microscope equipped with a plan Apochromat 40x/1.3 Oil DIC M27 (Zeiss, Oberkochen, Germany) objective, using a gallium arsenide phosphide photomultiplier tube (GaAsp-PMT) detector controlled by Zen black software (version 8.0.7.273, Zeiss, Oberkochen, Germany). After acquisition, images were processed using the Fiji ImageJ2 software (National Institutes of Health, Bethesda, MD, USA).

### 2.8. Statistical Methodology

Statistical analysis was performed using the GraphPad software (version 10.2). All information regarding the tests used for statistical analysis is reported in Tables and Figures.

## 3. Results

### 3.1. TNF-a Increases in the Plasma of Patients with SCI and AD

In total, 34 HLT volunteers and 99 patients (30 with SCI, 30 with MCI, and 39 with AD) were enrolled in this study. A homogeneous number of men and women were included with no significant discrepancies in their educational backgrounds. Patients with AD had lower MMSE scores than HLT controls and patients with SCI or MCI. No differences in BMI were observed in the HLT, SCI, MCI, and AD groups. In terms of comorbidities, the two groups were fairly homogeneous, although 8 out of 39 patients with AD had diabetes (Table 1). Notably, a significant negative correlation was observed between the participants’ age and education level (r = −0.3, *p* = 0.004), BMI (r = −0.30; *p* = 0.002), and MMSE score (r = −0.40; *p* = 0.00004).

**Figure 1:**
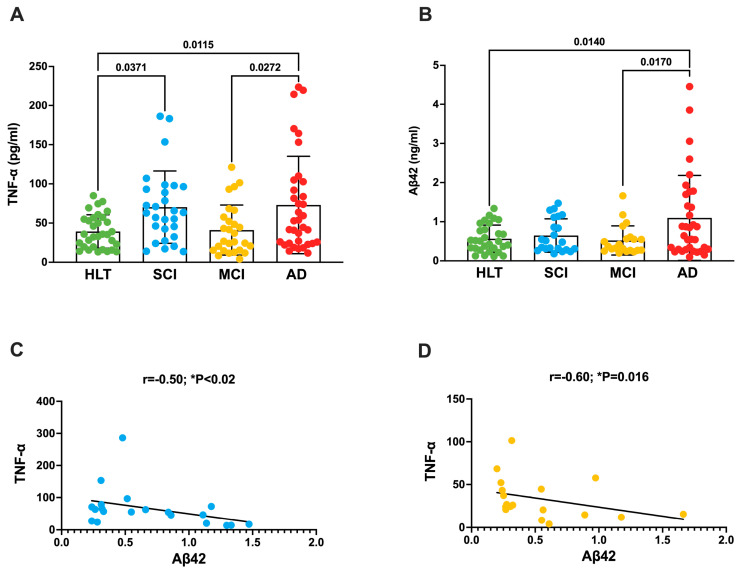
Plasma levels of TNF-α and Aβ42 increase in patients with AD. (**A**) TNF-α and (**B**) Aβ42 levels in the plasma of patients with SCI (blue dots), MCI (yellow dots), and AD (red dots), and in HLT (green plots) controls. Data below the threshold of the ELISA kits were excluded from the analysis. One-way analysis of variance followed by Tukey’s test for multiple comparisons was used to evaluate statistical differences. The *p* values are reported in the figure. Spearman’s correlation analysis between TNF-α and Aβ42 levels in the plasma of patients with SCI (**C**) and MCI (**D**). (r) = Spearman’s correlation coefficient. *r* values higher than ±0.50 were considered significantly different. Statistical significance was set at *p* (*) < 0.05. TNF-α correlation analyses vs. age, BMI, MMSE, or Aβ42 are presented in Table 2.

Initially, we determined the plasma levels of TNF-α in all participants. Our findings showed that TNF-α levels were higher in patients with SCI and AD than in HLT controls (Figure 1A and Table 1). Interestingly, the TNF-α levels in patients with AD were also higher than those in patients with MCI.

In parallel, we evaluated the amount of Aβ42 in the plasma of all four groups (Table 1) and we found that Aβ42 levels were significantly higher in patients with AD than in those with MCI and in HLT controls (Figure 1B). Notably, both TNF-α and Aβ42 levels were lower in the patients with MCI than in those with AD.

Next, we analyzed the correlations between TNF-α, Aβ42, and MMSE scores with respect to the four experimental groups: HLT, SCI, MCI, and AD. We found that TNF-α and Aβ42 levels were negatively correlated in patients with SCI (r = −0.50) and MCI (r = −0.60) (Figure 1C,D), but not in those with AD or in HLT volunteers (Table 2). Similarly, we did not observe correlations between TNF-α and age, BMI, or MMSE scores, which are all considered risk factors for AD (Table 2) [22,23].

### 3.2. TNF-α Does Not Initiate Amyloidogenic Processes in Human Microglial Cell Cultures

**Figure 2:**
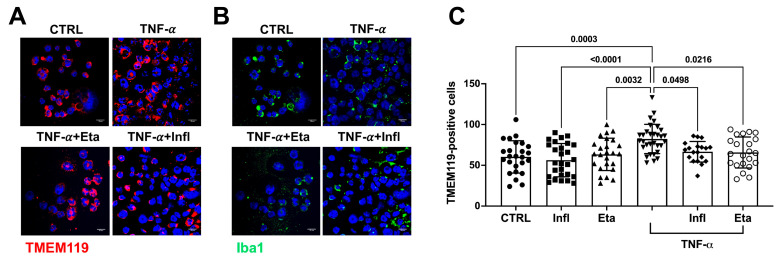
TNF-α promotes neuroinflammatory processes and induces microglial proliferation. Representative pictures of (**A**) TMEM119 (red) and (**B**) Iba1 (green) immunostaining in microglial cells (CTRL) incubated with TNF-α (TNF-α, 120 ng/mL) in the presence or not of Eta, 1 μg/mL, or Infl, 50 μg/mL. The time course of cell growth in the presence or absence of TNF-α is shown in Figure S2. Nuclei were stained with DAPI (blue). Scale bar: 15 µm. 20× objective. The number of TMEM119-positive nuclei was counted, and the quantitative differences are shown in (**C**). Dot symbols represent the number of nuclei counted in each field. Experiments were performed three times in triplicate. Significance among the groups was calculated by one-way ANOVA followed by Tukey’s test for multiple comparisons and expressed as *p* values.

We then analyzed the activation of the inflammatory pathway in human microglia exposed to TNF-α for 48 h. TNF-α incubation increased the number of DAPI-positive nuclei (Figure S1A). The same increase was also evident when we counted the number of cells positive for TMEM119 staining, which is used as a microglial cell marker [33] (Figure 2A). Of note, TNF-α inhibitors, Eta and Infl, were able to block both cell proliferation (Figure S1B) as well as the number of TMEM119-positive cells (Figure 2C) after TNF-α incubation at the concentrations of 1 μg/mL and 50 μg/mL, respectively. The same TMEM119-positive cells were also stained with Iba-1, a protein expressed in microglial cells that is upregulated during inflammation (Figure 2B) [33].

**Figure 3:**
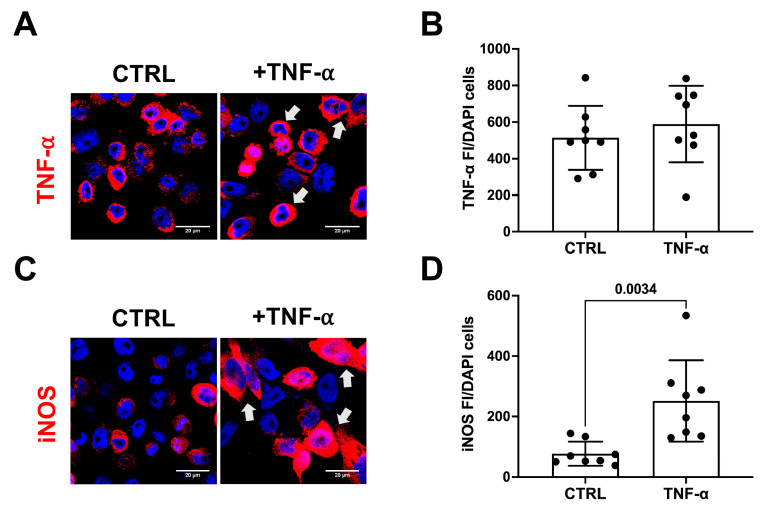
TNF-α induces iNOS expression in microglial cells. (**A**) Representative pictures of TNF-α (**A**) and iNOS (**C**) immunostaining in microglial cells incubated with TNF-α (+TNF-α, 120 ng/mL) or with vehicle (CTRL). Nuclei were stained with DAPI (blue). Arrows mark cell aggregates. Scale bar: 20 µm, 40× objective. (**B**,**D**) show the immunofluorescence intensity (FI) analysis of TNF-α and iNOS, respectively, calculated as the ratio between the mean intensity and the number of DAPI-positive cells. The experiments were performed two times in quadruplicate. The data are expressed as mean ± SEM. Only *p* values < 0.05 are reported.

In addition, we observed a large increase in the number of iNOS-positive cells 48 h after TNF-α exposure (Figure 3A), in line with previous studies demonstrating that TNF-α amplifies and exacerbates the inflammatory response in microglial cells [17]. Notably, cells exposed to TNF-α appeared to have a smaller size (Figure 3A,C) and aggregated in large clusters (see arrow).

**Figure 4:**
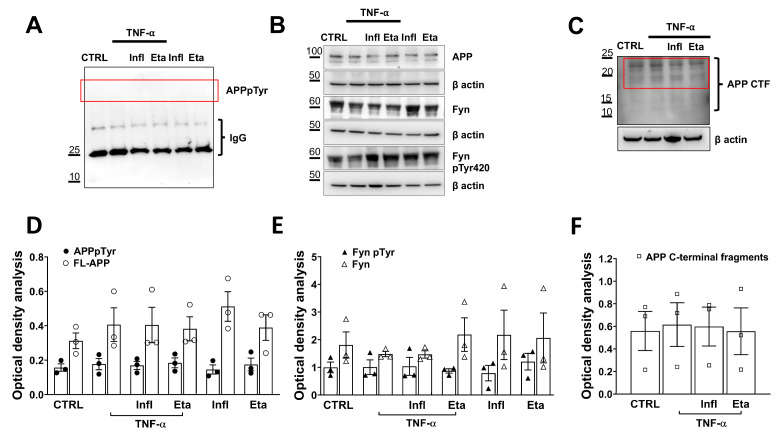
TNF-α does not activate amyloidogenic processes. (**A**) Representative WB analysis of APPpTyr682 (red box) and (**B**) APP, Fyn, and phosphorylated Fyn levels, as well as the corresponding β actins, in microglial cells 48 h after TNF-α (120 ng/mL) exposure 24 h after TNF-α exposure cells were exposed to Eta (0.5 μg/mL) or Infl (50 μg/mL) and left in culture for additional 24 h. The WB optical density (OD) analyses are reported in (**D**,**E**). (**C**) WB analysis for APP C-terminal fragment. (**F**) OD analysis of APP-C terminal processing refers to the pattern of bands migrating approximately 20 kDa as suggested in previous studies [36] (red rectangle). Significance among the groups was calculated by one-way ANOVA followed by Tukey’s test for multiple comparisons and expressed as *p* values. *p* values higher than 0.05 were considered not significant and were not reported in the graph.

Next, we evaluated whether TNF-α-related inflammatory processes affected amyloidogenesis in microglial cells. We assessed Aβ42 secretion levels in the media and found that they were below the threshold of ELISA kit sensitivity, both in the presence and absence of TNF-α. Therefore, we examined the intracellular molecular changes that preceded Aβ42 secretion, such as APPpTyr682 [27], which we previously reported to be potentially relevant as an earlier signature of AD [34]. We found that neither TNF-α alone nor in combination with the two inhibitors influenced APPpTyr682 levels at 48 h after incubation (Figure 4A,D). Consequently, the intracellular amyloidogenic APP processing was not affected by TNF-α exposure (Figure 4C,F), and full-length APP levels did not change (Figure 4B,E). As an additional marker of amyloidogenesis, we measured Fyn phosphorylation levels, which have been reported to be hyperphosphorylated in patients with AD [28] and trigger phosphorylation of both APPpTyr682 [27] and tau proteins [35]. As for APP, we did not detect differences in Fyn phosphorylation levels in microglial cells exposed to TNF-α when compared to untreated controls (Figure 4B,E), likely implying that TNF-α does not activate the amyloidogenic pathway per se, but most likely requires other factors that contribute to the process.

## 4. Discussion

The role of TNF-α in AD has recently garnered significant attention [37]. Elevated levels of TNF-α have been detected in the CSF of patients with AD [38] and around Aβ plaques in postmortem brains [39]. Intriguingly, the TNF-α G308A polymorphism exhibits a differential association with the risk of AD in Chinese and northern European populations, with a potentially increased risk in the former and a potentially decreased risk in the latter [10]. These results are further supported by evidence that TNF-α can pass through the intact blood–brain barrier (BBB), so therapies that lower systemic levels of this cytokine will indirectly reduce TNF-α levels in the brain [40]. In addition, several studies have described increased BBB permeability in patients with AD, implying that systemic TNF-α can largely affect brain function [41,42]. Zhou et al. demonstrated that patients with rheumatoid arthritis (RA) have a lower risk of AD, thus theorizing that therapies used to reduce inflammation in patients with RA might also protect patients with AD [9]. Consistently, Zheng et al. provided evidence regarding the association of two TNF-α inhibitors, adalimumab and etanercept, with a lower dementia risk in patients with RA over the 20-year study period [43]. However, despite these encouraging results, the outcomes of most studies on the use of TNF-α inhibitors in AD have been inconclusive, making it uncertain whether the use of these medications offers any benefits in the treatment of AD [44,45].

In this study, we analyzed TNF-α and Aβ42 levels in the plasma of patients with SCI, MCI, and AD, and observed that in addition to TNF-α and Aβ42 levels being higher in patients with AD, a correlation between these two signatures was only detectable in patients with SCI and MCI. This might suggest that TNF-α and Aβ42 are related to each other at a very early stage of the pathology and that this correlation is lost later in the disease when other factors further exacerbate Aβ-related signs and accelerate AD progression. In addition, we noted that patients with MCI showed significantly lower TNF-α and Aβ42 levels than those with AD, indicating that changes in these two signatures might characterize the transition from MCI to AD, and likely identify clinical profiles with the worst outcome of AD.

In this regard, it has previously been found that patients with systemic inflammatory events and elevated plasma TNF-α levels show a 10-fold higher rate of cognitive decline over a 6-month observation period [46,47]. Furthermore, these patients have a 2-fold increased likelihood of experiencing symptoms commonly associated with AD, such as apathy, anxiety, depression, and agitation [48].

We next evaluated whether TNF-α is involved in the activation of the amyloidogenic pathway. Previous studies carried out in patients with AD have suggested that TNF-α is independent of changes in Aβ42. Increased Aβ42 levels in patients with lower MMSE scores were correlated with altered plasma lysophosphatidylcholine levels rather than TNF-α levels. In addition, APPpTyr682 and Fyn phosphorylation increased in the peripheral blood mononuclear cells (PBMCs) of patients with high lysophosphatidylcholine, Aβ42, and TNF-α levels, thus making it apparent that the activation of the amyloidogenic pathway was more likely linked to multiple factors and affected by comorbidities of patients [21]. Indeed, the fact that patients involved in the study suffered from several different age-related pathologies made it difficult to discriminate which components in their plasma might have led to the changes in the Aβ42-related pathways.

In this regard, we should mention that TNF-α secretion increases in age-related pathological processes, such as atherosclerosis, an inflammatory disease that becomes more prevalent with age and pathological conditions, including those caused by viral or subclinical bacterial infections [19,20,21,22]. TNF-α causes alterations in specific metabolic pathways, including obesity [33] and hypercholesteremia, which worsen and accelerate the progression of AD [29,30,31]. TNF-α is involved in lipid metabolism, altering lipid storage and mobilization [49,50], and causing lipid peroxidation, which ultimately causes ROS production, overt ER stress, and exacerbates neuronal degeneration in patients with AD [51].

To exclude all of these confounding factors, which are often present in the clinical background of patients with AD, we analyzed the effect of TNF-α in human primary microglial cells. Compared to blood mononuclear cells, which we used in previous experiments, human microglial cultures offered the advantage of exhibiting properties similar to those of primary cultures without being affected by the individual clinical profiles of patients. We observed that microglial cells did not affect amyloidogenesis after 48 h of incubation, as assessed by increased APPpTyr682 levels, APP intracellular processing, and Fyn phosphorylation, which are responsible for the initiation of AD or AD-related pathologies [34,52]. However, microglial cells activated an inflammatory response upon TNF-α exposure, which was consistent with increased cell proliferation and iNOS expression. This result is in line with previous evidence demonstrating that TNF-α activates an inflammatory pathway in microglial cells and triggers oxidative stress reactions by increasing iNOS expression and NO secretion [53,54]. This evidence also underlines an additional putative role that TNF-α might play in the progression of AD, triggering oxidative stress and exacerbating already activated neurodegenerative processes.

Overall, it is likely that changes in TNF-α levels result in widespread inflammatory processes that can impact cell functions with multiple mechanisms ranging from the lipid peroxidation to a progressive increase in reactive oxidative species secretion, affecting either peripheral blood cells or the brain population. When TNF-α levels increase and Aβ42 levels decrease, as observed in individuals with SCI and MCI, this may lead to more severe outcomes and accelerated progression towards AD. However, this likely implies that TNF-α exacerbates amyloidogenesis when the latter is initiated.

## 5. Conclusions

This study posits that TNF-α acts independently of amyloidogenesis, although it influences and exacerbates it in the initial stages of the disease by eliciting inflammatory-related processes. Interestingly, TNF-α levels were higher in patients with SCI, and TNF-α and Aβ42 levels were negatively correlated in patients with SCI and MCI. This finding reinforces the interest in TNF-α as an early signature in patients at high risk of developing AD and suggests that it may represent a signature predictive of a worse outcome of AD progression if analyzed alongside Aβ42 levels. Indeed, it would be valuable to monitor patients with SCI and higher TNF-α levels over time, with respect to those with no changes in TNF-α levels, to determine the impact of this inflammatory response on the progression of AD or other pathologies.

## Figures and Tables

**Table 1:** Clinical features of subjects included in the study. Data are expressed as mean ± standard error of the mean (SEM). Comparisons of the patient clinical characteristics were analyzed using the chi-square test or unpaired T test. Statistical significance was set at *p* < 0.05. Mean values of TNF-α and Aβ42 levels are reported in the four experimental groups. Significance among the groups was calculated by one-way ANOVA followed by Tukey’s test for multiple comparisons and expressed as *p* values. (*) TNF-α mean values from SCI and AD groups are significantly higher than those from HLT; (^§^) TNF-α mean values in patients with AD are higher than those in patients with MCI; (^#^) Aβ42 mean values in patients with AD are higher than those in patients with MCI and HLT subjects.

	HLT (n.34)	SCI (n.30)	MCI (n.30)	AD (n.39)	Chi-Square Test or Unpaired T Test (*p* Value)
**Gender**	15F/19M	17F/13M	18F/12M	20F/19M	NS
**Age** **(years, Mean ± SEM)**	67.9 ± 1.5	71.2 ± 1.2	68.2 ± 1.9	78.4 ± 1.3	NS
**Education** **(years, Mean ± SEM)**	10.7 ± 0.7	11.7 ± 0.9	10.7 ± 0.9	7.97 ± 1.0	NS
**BMI** **(Mean ± SEM)**	28.5 ± 0.73	27.1 ± 0.8	27.6 ± 0.7	25.2 ± 0.8	NS
**MMSE** **(Mean ± SEM)**	29.55 ± 0.31 *	29.8 ± 0.3 *	27.9 ± 0.3 *	10.7 ± 1.3	* (*p* < 0.05 vs. AD)
**Risk factors**					
Smokers	19/34	11/30	9/30	11/39	NS
Alcohol (more than 3 glasses)	0/34	1/30	2/30	4/39	NS
**Comorbidities**					
Hypertension	18/34	18/30	16/30	17/39	NS
Myocardial infarction	1/34	1/30	0/30	3/39	NS
Tia/stroke	0/34	0/30	2/30	1/39	NS
Dyslipidemia	8/34	10/30	11/30	9/39	NS
Diabetes	* 0/34	2/30	3/30	8/39	* (*p* = 0.04 vs. AD)
**TNF-α**	39.30 ± 3.8	73.9 ± 10.9 *	41.0 ± 6.15 ^§^	73.0 ± 10.5 *	* *p* < 0.05 vs. HLT ^§^ *p* < 0.05 vs. AD
**Aβ42**	0.57 ± 0.063 ^#^	0.65 ± 0.09	0.52 ± 0.08 ^#^	1.09 ± 0.18	^#^ *p* < 0.05 vs. AD

**Table 2:** Spearman’s correlation analysis between TNF-α and age, BMI, MMSE, or Aβ42 in the HLT, SCI, MCI, and AD groups. r = Spearman’s correlation coefficient; p = *p* value; n = number of correlation pairs, CI = confidence interval. The XY correlation plots of TNF-α vs. Aβ42 in patients with SCI and MCI are reported in Figure 1C,D.

**TNF-α vs.**		**HLT**	**SCI**	**MCI**	**AD**
	**Age**				
	r	0.04	−0.21	−0.02	0.05
	p	0.82	0.28	0.9	0.77
	95% CI	−0.3 to 0.4	−0.5 to 0.2	−0.4 to 0.4	−0.3 to 0.3
	n	32	28	27	35
	**BMI**				
	r	0.17	0.08	−0.17	−0.16
	p	0.35	0.65	0.39	0.39
	95% CI	−0.2 to 0.5	−0.3 to 0.5	−0.5 to 0.2	−0.5 to 0.2
	n	32	28	27	29
	**MMSE**				
	r	−0.12	−0.11	0.16	0.13
	p	0.52	0.57	0.41	0.45
	95% CI	−0.4 to 0.2	−0.47 to 0.28	−0.2 to 0.5	−0.2 to 0.5
	n	32	28	27	35
	**Aβ42**				
	r	−0.30	−0.50	−0.60	−0.20
	p	0.17	0.03	0.016	0.32
	95% CI	−0.6 to 0.1	−0.8 to 0.05	−0.8 to −0.1	−0.5 to 0.2
	n	29	20	18	32

## Data Availability

Data will be available upon request. The original files of all WBs shown in the manuscript have been uploaded during submission.

## Supplementary Materials

The following supporting information can be downloaded at: https://www.mdpi.com/article/10.3390/antiox13020216/s1, Figure S1: Curve–dose effects of TNF-α and TNF-α inhibitors. Cells were treated with TNF-α at concentrations ranging from 80 ng/mL to 240 ng/mL for 48 h (**A**). The nuclei were stained with DAPI and counted. The lowest concentration of TNF-α (120 ng/mL) that was able to increase cell proliferation without activating toxic processes was selected and used in all other experiments. After 24 h of TNF-α incubation, the cells were washed and incubated with etanercept (Eta, 1 µg/mL) and infliximab (Infl 50 µg/mL) for an additional 24 h (**B**). As the two inhibitors did not affect cell survival, we used Eta and Infl at concentrations suggested by the supplier, such as 1 µg/mL and 50 µg/mL, respectively (**C**,**D**). The experiments were repeated thrice in triplicate. Dots represent each field counted on the slides. One-way ANOVA followed by Tukey’s test for multiple comparisons was used for statistical analysis. Statistical significance was set at *p* < 0.05. *p* values < 0.05 were reported for each graph; Figure S2: Time course of TNF-α exposure in microglial cells. The figure shows three different experimental settings. (**A**) Control condition, in which cells were grown for 24 h and 48 h with the vehicle (PBS) in the absence of TNF-α. Note that t0 refers to cells 24 h after plating. Cells were stained with microglial marker TMEM119 (red) and nuclei with DAPI (blue). (**B**) Single-exposure condition, in which cells were exposed to TNF-α at a concentration of 120 ng/mL for 24 h (t24) or 48 h (t48). (**C**) Double-exposure condition, in which TNF-α was added twice, first at t0 and then at t24. The experiment was stopped 48 h from the first exposure. Scale bar: 15 µm (**D**) Nuclei stained with DAPI were counted and values were analyzed using one-way ANOVA followed by Tukey’s test for multiple comparisons. The experiments were repeated three times in triplicate. Symbols in the histograms refer to the number of cells counted on each field on the slides. *p* values < 0.05 were reported on the graph.
