# Supplementary material for: TNF-α Levels Are Increased in Patients with Subjective Cognitive Impairment and Are Negatively Correlated with β Amyloid-42
Source: Antioxidants (Basel). 2024 Feb 8;13(2):216. doi: 10.3390/antiox13020216 (PMC10886257; doi:10.3390/antiox13020216)
Supplement: Supplementary file 1 [file antioxidants-13-00216-s001.zip › antioxidants-2800363-supplementary.pdf]

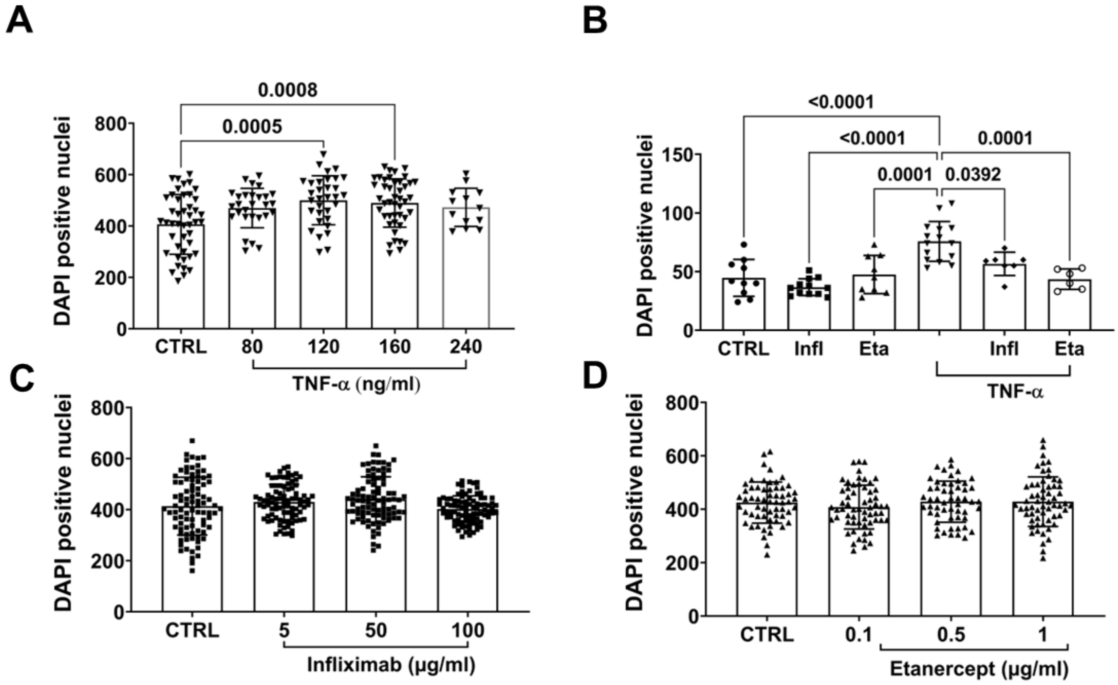

Figure S1. Curve-dose effects of TNF-α and TNF-α inhibitors. Cells were treated with TNF-α at concentrations ranging from 80 ng/mL to 240 ng/mL for 48 h (**A**). The nuclei were stained with DAPI and counted. The lowest concentration of TNF-α (120 ng/mL) that was able to increase cell proliferation without activating toxic processes was selected and used in all other experiments. After 24 h of TNF-α incubation, the cells were washed and incubated with etanercept (Eta, 1 µg/mL) and infliximab (Infl 50 µg/mL) for an additional 24 h (**B**). As the two inhibitors did not affect cell survival, we used Eta and Infl at concentrations suggested by the supplier, such as 1 µg/mL and 50 µg/mL, respectively (**C**,**D**). The experiments were repeated thrice in triplicate. Dots represent each field counted on the slides. One-way ANOVA followed by Tukey's test for multiple comparisons was used for statistical analysis. Statistical significance was set at $p < 0.05$. p values < 0.05 were reported for each graph.

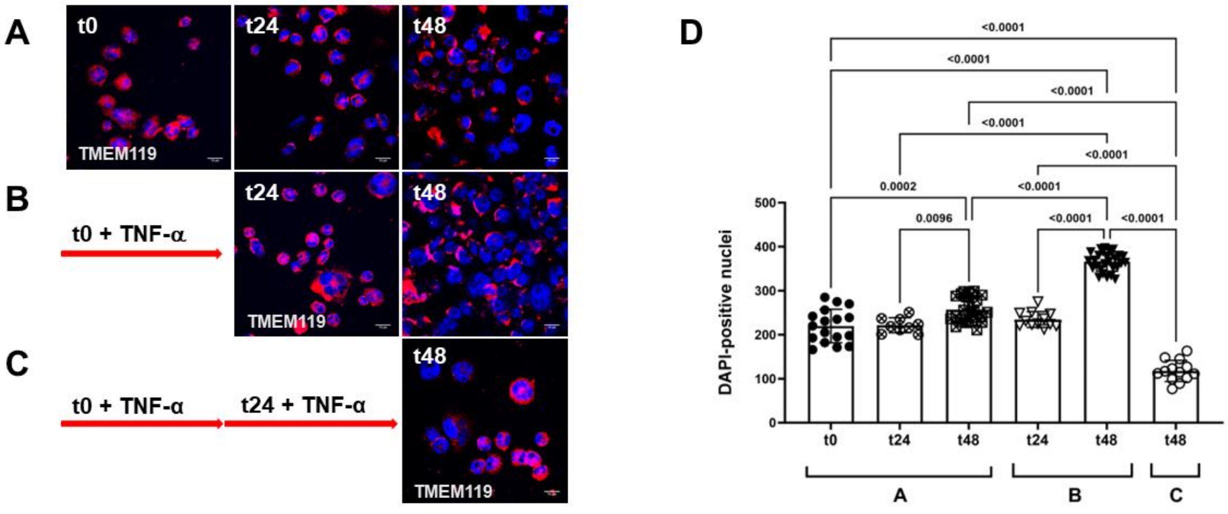

Figure S2. Time course of TNF-α exposure in microglial cells. The figure shows three different experimental settings. (**A**) Control condition, in which cells were grown for 24 h and 48 h with the vehicle (PBS) in the absence of TNF-α. Note that t0 refers to cells 24 h after plating. Cells were stained with microglial marker TMEM119 (red) and nuclei with DAPI (blue). (**B**) Single exposure condition, in which cells were exposed to TNF-α at a concentration of 120 ng/mL for 24 h (t24) or 48 h (t48). (**C**) Double exposure condition, in which TNF-α was added twice, first at t0 and then at t24. The experiment was stopped 48 h from the first exposure.

(**D**) nuclei stained with DAPI were counted and values were analyzed using one-way ANOVA followed by Tukey's test for multiple comparisons. The experiments were repeated three times in triplicate. Symbols in the histograms refer to the number of cells counted on each field on the slides. p values < 0.05 were reported on the graph.
